# Integrated hepatitis C treatment is associated with improved retention and success in outpatient treatment for opioid use disorder at a private clinic

**DOI:** 10.3389/fpsyt.2022.932306

**Published:** 2022-09-14

**Authors:** Phyllis Losikoff, Jordon D. Bosse, Stephen A. Martin, Amanda Wilson, Lisa M. Chiodo

**Affiliations:** ^1^CleanSlate Outpatient Addiction Medicine, New Bedford, MA, United States; ^2^Division of Pediatric Infectious Disease, The Warren Alpert Medical School of Brown University, Providence, RI, United States; ^3^School of Nursing, Bouvè College of Health Sciences, Northeastern University, Boston, MA, United States; ^4^Massachusetts General Hospital, Boston, MA, United States; ^5^Barre Family Health Center, Barre, MA, United States; ^6^Department of Family Medicine and Community Health, UMass Chan Medical School, Worcester, MA, United States; ^7^Addiction Research and Education Foundation, Gig Harbor, WA, United States; ^8^Elaine Marieb College of Nursing, University of Massachusetts, Amherst, MA, United States

**Keywords:** hepatitis C infection, opioid use disorder, direct acting antiretrovirals, integrated care, addiction medicine

## Abstract

**Background:**

Direct acting antiretrovirals (DAA) are effective for individuals who are infected with chronic hepatitis C virus (HCV), yet many people go without access to these lifesaving treatments.

**Materials and methods:**

We conducted a non-randomized study evaluating treatment data for patients in outpatient treatment for opioid use disorder (OUD) at a private clinic. Patients who were HCV-positive, had been in OUD treatment for at least 4 weeks, and engaged in integrated HCV treatment with DAA (co-located within their treatment for OUD) were compared to patients with HCV who only received OUD treatment. We evaluated HCV cure; OUD medication adherence, treatment utilization and retention; and illicit substance use for those engaged in treatment between 9/2016 and 1/2018.

**Results:**

Seventy-four patients completed integrated HCV-OUD treatment with DAA, with 87.8% achieving cure. Of the 66 who completed treatment and were subsequently evaluated for sustained viral response 98.5% were cured. Patients who received integrated HCV and OUD treatment in our clinic, stayed in OUD treatment longer, demonstrated higher OUD medication adherence, and used less opioids or cocaine compared to HCV-infected patients (*n* = 572) being treated only for OUD.

**Discussion:**

We have reported on a reproducible intervention that lends itself to outpatient OUD treatment. Analyses demonstrate the potential positive impact HCV treatment has on OUD recovery, including reduction in opioid and cocaine use and increased retention in care

**Conclusion:**

Co-locating HCV treatment with existing OUD treatment is feasible, effective, and demonstrates positive outcomes for the treatment of both conditions.

## Introduction

Chronic hepatitis C virus (HCV) infection is a major public health problem in the United States. A leading cause of cirrhosis and hepatocellular carcinoma, its mortality rate has been estimated at over 35% (1). The majority of the estimated 3.5 million Americans with chronic infected HCV are unaware of their infection (2). The current primary risk factor for HCV acquisition is injection drug use; 84% of cases with risk factor data in 2014 were among persons who inject drugs (PWID) (3). Increases in injection drug use have led to a 6-fold increase in new cases of HCV among persons 20–39 years old, from 2004 to 2018 (4). Morbidity and mortality associated with HCV proportionately impacts the lives of current and former PWID.

Direct acting antiviral (DAA) regimens for chronic HCV are safe and efficacious (5). They have revolutionized our ability to cure patients with chronic HCV infection. In addition to prevention of transmission and progression of liver disease, patients cured of HCV have decreased risk of diabetes, stroke, and improved cognitive function (6–8). Successful DAA therapy is also associated with significant improvements in objective and subjective measures of quality of life (9, 10). Despite the availability of DAA, the majority of PWID are not being treated (11) and only a small percentage know that newer treatments are highly effective with few side effects (12). Even when many patients in opioid use disorder (OUD) treatment are screened, it can be difficult to arrange successful treatment (11). Identifying and implementing interventions that reduce or eliminate barriers to HCV treatment is critical if we are to achieve the World Health Organization goal of eliminating HCV globally by 2030 (13).

PWID and their HCV infections, inextricably linked, are stigmatized and discriminated against, resulting in mistrust of medical providers and establishments (14–16). Patients and providers alike harbor misperceptions about whether abstinence is needed for treatment (17, 18). Initiatives aimed at overcoming these barriers in order to screen and treat PWID are vital in both treating the individual patient and reducing further transmission to stem this epidemic (19, 20). National organizations support treatment of PWID, with or without active substance uses (5, 21). Studies support high rates of sustained virological response (SVR) in people who are in recovery with opioid agonist treatment (17, 22) and those who are not, including those with active opioid use (23).

Co-location of HCV treatment together with addiction treatment, as recommended by the American Society of Addiction Medicine, eliminates additional referral (24). Patients are cared for by a trusted provider, avoiding potential stigma. We have begun integrating HCV treatment in our outpatient treatment for OUD services (25). To our knowledge, we are among the first commercial outpatient OUD treatment clinic to provide HCV treatment directly to patients in OUD treatment rather than referring them to primary or other specialist care. The purpose of this analysis is to evaluate the relationships between integrated HCV treatment on patients' HCV status and their OUD treatment.

## Materials and methods

### Study design

We conducted a non-randomized study using de-identified data from the electronic health record of patients being treated with medication for opioid use disorder (MOUD) in an outpatient treatment program in New Bedford, MA. Patients who received integrated treatment for their chronic HCV were compared with those who have not yet been treated to examine recovery outcome measures: treatment retention and utilization, medication adherence, and substance use. This research was reviewed by the Institutional Review Board at Northeastern University and deemed as not human subjects' research.

### Setting

CleanSlate Outpatient Addiction Medicine, is a national company of free-standing, outpatient addiction treatment centers. CleanSlate Outpatient Addiction Medicine provides medication treatment for disorders of opioid and alcohol use. All patients initiating substance use treatment are screened for blood-borne pathogens at their initial visit including Hepatitis B virus, HIV, and HCV testing with reflex HCV ribonucleic acid polymerase chain reaction and genotyping for all HCV antibody-positive specimens. All [Treatment Program] providers are trained in pre- and post-test counseling, including educating patients about HCV DAA therapy.

### Intervention

Beginning on 9/1/2016, patients with evidence of chronic HCV (i.e., viremic) who were adherent to their OUD treatment, operationalized as attending scheduled clinic visits with a medical provider, for at least 4 weeks (to increase probability of adherence to HCV medication, abstinence from illicit substances was not required) were offered HCV treatment on site at CleanSlate Outpatient Addiction Medicine in New Bedford, MA. Exclusion criteria included co-infection with HIV or hepatitis B, or unstable psychiatric disorder that would impair a patient's ability to adhere to the regimen. Interested patients were evaluated, those with signs or symptoms of decompensated cirrhosis or significant drug-drug interactions were referred to a hepatologist for treatment. Eligible candidates were started on DAA treatment by an advanced practice nurse who was trained to provide HCV treatment to existing OUD patients. The practitioner completed 40 h of online training and one in-person training session (8 h) with an infectious disease specialist. Throughout treatment, supervision was provided by an addiction medicine/infectious disease specialist. All treatment regimens were determined in accordance with guidelines approved by the American Association for the Study of Liver Diseases and the Infectious Diseases Society of America (5). The patients returned for one additional visit once their medications arrived at the clinic, to initiate treatment, during which side effects and the importance of adherence was reviewed. Once DAA treatment was initiated, patients' course of HCV care was monitored during their standard OUD visits.

### Sample

The treatment group consisted of 74 patients (71.6% men) with HCV who received treatment with DAA as part of their treatment for OUD at an office-based medication treatment program. They are being compared to 572 other patients with chronic HCV who were receiving outpatient treatment with MOUD at the same office-based treatment program. The control group met the inclusion criteria for the intervention (viremic, without HBV or HIV, and attended OUD treatment visits with their provider for at least 4 weeks), but did not have a visit with the HCV NP during the period of the pilot either by choice or limited capacity of a single provider.

### Study outcomes

To examine the relationships between integrated OUD/HCV treatment intervention on both HCV and OUD outcomes, differences between the two groups were evaluated. Response to HCV treatment was determined by HCV viral load 12-weeks post completion of treatment, sustained viral response (SVR).

Outcomes included OUD treatment utilization and retention, and substance use. Several variables were used to measure OUD treatment utilization: total time in care, time since the last visit, number of maintenance visits, and number of “re-join visits.” If the time since the last visit was substantial, the patient might also be required to “re-join” the program. According to OUD treatment protocol, patients were required to come to the clinic for scheduled maintenance visits. Treatment retention was defined as retained or not retained (dichotomous yes/no) based on the patient's status as of January 2018.

To measure medication utilization, the total number of urine drug screens, which are performed at each clinic visit, frequency of which is determined by patients' stage of recovery, that were positive for buprenorphine was divided by the total number buprenorphine urine drug screen tests performed for each patient. The value resulted in the percentage of positive buprenorphine tests. Higher values indicated higher rates of appropriate medication utilization. To measure substance use, a similar percent positive value was obtained for urine drug screens results from each of the following drug categories: alcohol, amphetamines, benzodiazepines, cocaine, all other illicit opioids, and THC.

### Data analysis

Patient characteristics between groups were compared using a paired *t-*test (age) and chi square analysis (gender). Between group differences of retention in treatment was evaluated using logistic regression. Between group differences in all remaining continuous outcomes were measured by ANCOVA (univariate, general linear model). Patient age and gender were included as covariates in all models. Race and ethnicity were not included as covariates in the regression model due to missing data (race % missing = 30.7; ethnicity % missing = 24.5). Available data indicates the sample is homogenous (94.4% White; 88.5% non-Hispanic). All analyses were conducted in SPSS v. 25 (26).

Because an individual who was in care for a longer duration would have higher values for many of the variables examined, regardless of care engagement, the treatment utilization variables and number of rejoin and maintenance visits were adjusted by the total time in care. Total time in care was measured as all time as represented in the electronic medical record. This included from each patient's initial visit to the end of their care episode or the end of the data collection time.

## Results

### Patient characteristics

All patients included in the analyses (*N* = 646) met criteria for HCV treatment; 74 received the OUD/HCV integrated treatment intervention. Mean patient age for all participants was 39.0 (*SD* = 10.2, range = 21.0–67.9). There was no difference in age (*t* = −1.6, *df* = 643, *p* = 0.203) or gender (χ^2^ = 2.8, *df* = 1, *p* = 0.094) between groups.

### HCV results

**Figure 1:**
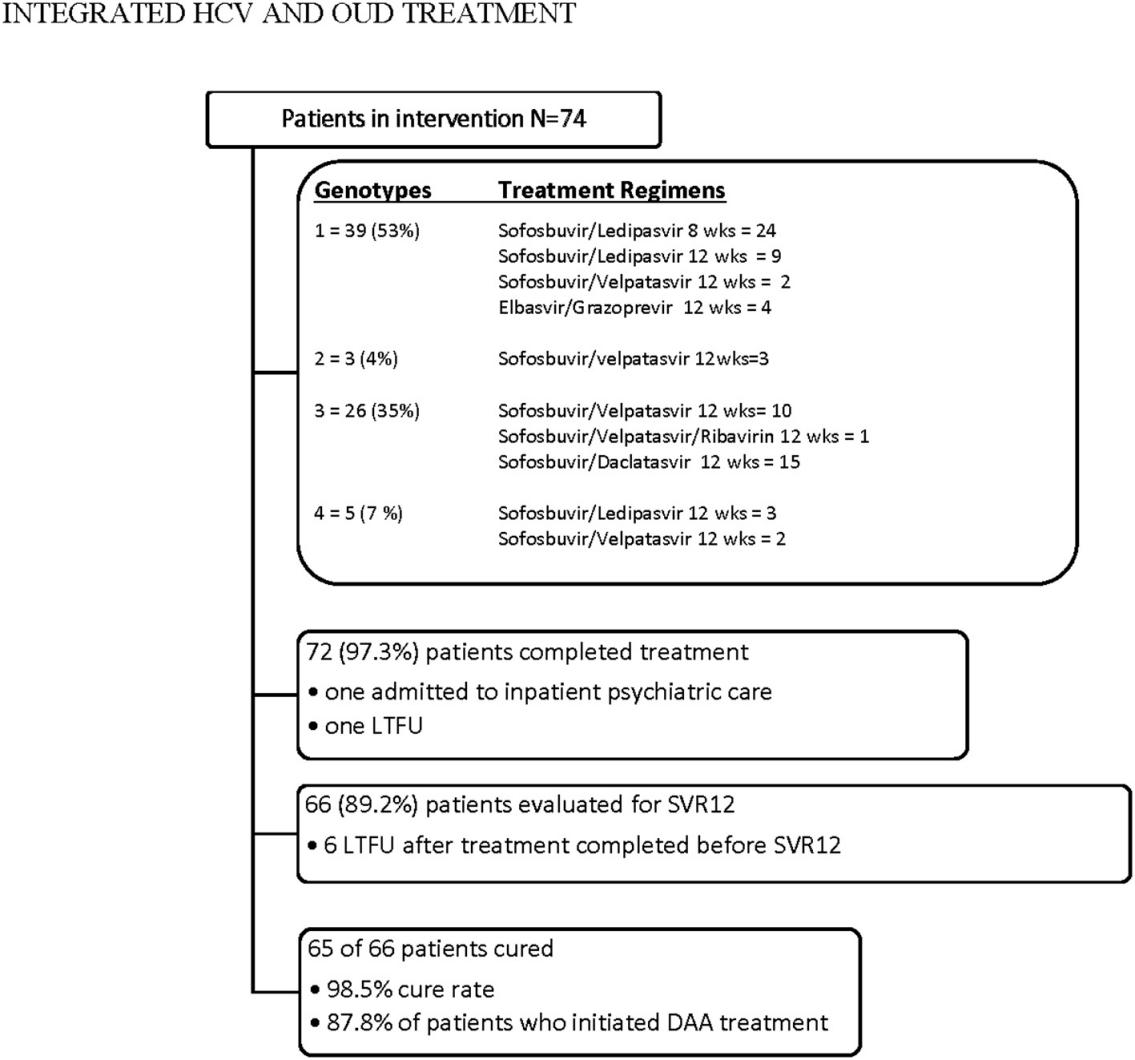
Patient treatment flow.

Over half the patients treated for HCV were genotype 1 (55.4%, *n* = 41) and about a third (33.8%, *n* = 25) were genotype 3. The remainder were genotype 4 (8.1%, *n* = 6) or 2 (2.7%, *n* = 2). Figure 1 highlights patients' progression through treatment. Nearly all (97.3%) who initiated DAA treatment completed treatment. Six of the patients who completed treatment were lost to follow-up prior to SVR assessment. Among the 66 patients for whom SVR post-treatment data are available, all but one (98.5%) achieved cure. When considering all patients who initiated DAA treatment, 87.8% (*n* = 65*)* achieved cure. No patients in the treatment group stopped HCV treatment due to side effects or adverse effects.

### OUD results

Patients receiving integrated HCV treatment (treatment) had higher rates of OUD treatment utilization, attended more OUD maintenance visits, and were in OUD treatment for a longer duration than patients receiving MOUD alone (control). Patients in the treatment group had less time since their last visit and fewer rejoin visits than those in the control group. There was no significant difference in adherence to buprenorphine for those in treatment group compared to the control group.

### Substance use

After adjusting for covariates (age and gender), patients in the intervention group had lower rates of opioid and cocaine use. Notably, there was no difference in alcohol, THC, benzodiazepine, or amphetamine use (See Table 1).

**Table 1:** OUD treatment utilization, medication utilization, and substance use by treatment group^a^.

	**Treatment group**					
	**Control**		**Intervention**			
	**Mean**	**Standard error**	**Mean**	**Standard error**	***F***	***p***
**Treatment utilization**
Total time in care (years)	1.0	0.05	2.1	0.13	67.2	<0.001
Time since last visit (years)	1.1	0.03	0.3	0.09	74.9	<0.001
# rejoin visits	0.7	0.04	0.4	0.12	4.8	0.029
# maintenance visits	30.7	0.69	37.2	2.00	9.4	0.002
**Medication utilization** ^†^
Buprenorphine	82.2	1.1	87.5	3.2	2.4	0.119
**Substance use** ^†^
Alcohol	14.3	1.0	15.2	2.2	0.1	0.770
Amphetamines	5.0	0.6	5.9	1.8	0.2	0.637
Benzodiazepines	11.3	0.8	11.9	2.3	0.6	0.809
Cocaine	20.8	1.2	10.1	3.3	9.7	0.002
Opioids	14.6	0.7	5.2	1.9	21.1	<0.001
THC	34.5	1.7	28.5	4.6	1.5	0.222

^a^All analyses control for age and gender.

^†^Percent positive tests.

### Treatment retention

Analyses were performed to examine the relationship between intervention groups and patient retention. A patient was considered retained if they were a patient at the end of the data collection period. The logistic regression model predicting treatment retention was significant (*p* <0.001) and 18.2% of the variance was explained (Nagelkerke *R*^2^). The relationship between the intervention groups and treatment retention was statistically significant after covariate control (*B* = 2.6, *p* < 0.001). Patients who received integrated OUD/ HCV treatment were 13.4 times (OR; 95% CI = 7.1–25.3) more likely to be retained in care with MOUD than patients who were not in the intervention group. Neither gender nor age were significantly related to retention (*B* = 0.3, *p* = 0.174 and *B* = 0.01, *p* = 0.456, respectively).

## Discussion

Despite carrying the highest burden of HCV infection, fewer than 10% of PWID evaluated for HCV subsequently initiate treatment (17). Barriers to diagnosis and treatment of PWID impede linkage to care (27, 28). Our analyses demonstrate that the integration of HCV diagnostic and treatment services in outpatient OUD treatment overcomes some of the barriers and stigma that have historically impeded PWID accessing and completing treatment. Consistent with reported literature, when receiving HCV treatment integrated with MOUD, patients achieve SVR (cure rates) comparable to non-drug users (17, 28). Our setting, a commercial clinic for outpatient treatment of OUD, is novel, and addresses the need for treatment settings to provide more comprehensive services (29), including HCV testing. To our knowledge we are the first in the US to report integration of HCV care within this setting. This innovative approach broadens access to HCV treatment for this heavily affected yet underserved population, and has the potential to reduce morbidity and mortality, decrease health care expenditures, and stem transmission among high-risk individuals (9, 19, 20). Our evidence also builds on prior literature of high HCV cure rates with co-located HCV treatment provided in other opioid treatment programs (e.g., methadone clinics) (28, 29).

Non-clinical benefits for patients cured of HCV include improved quality of life and sense of wellbeing (30). Patients with OUD treated in the interferon era reported reduction in substance use-and return to “normality” as a result of curing their HCV (30, 31). Our analyses of patients with OUD who received integrated care with DAA in our clinic, builds on this literature, providing an objective assessment of measures of utilization of addiction care and indices of recovery. Notably, patients treated for HCV in our clinic were more adherent to all visit types.

We observed lower rates of opioid and cocaine use in the intervention group. However, despite the trend toward improved buprenorphine adherence in the intervention group, the difference was not significant, suggesting that the decline in use of illicit opioids and cocaine was not solely attributable to better buprenorphine utilization, which is consistent with prior literature (31). The significantly lower rate of cocaine use in the intervention group may be related to the decreased chronic fatigue and increased energy patients cured of HCV report (32), removing the need for cocaine's stimulating qualities. Prior qualitative work suggests being cured of HCV may alleviate internal stigma and catalyze improved self-care, resulting in reduced opioid and cocaine use (31). One patient in this sample cured of HCV told their provider PL,

“… *It's out of my body. I don't feel dirty anymore. I feel better, like I've accomplished a big step in my recovery … getting rid of the past. It's the last part of the guilt and stigma around injecting.”*

Especially encouraging is the significant association of HCV treatment with higher utilization and better retention in OUD treatment when controlling for time in care. Previous research has identified HCV infection as factor impeding long-term treatment retention (33). Engaging and retaining patients in treatment with MOUD decreases mortality and is associated with a more durable and safer recovery (34). Improved retention in the treatment group could be due to pre-existing clinical relationships based on trust, which may act as an antidote to the sequelae of stigma (33). It is also possible that providing HCV treatment was the mechanism that engendered trust and a stronger connection, resulting in increased retention.

### Limitations

Inclusion and exclusion criteria (e.g., being stable in care for 4 weeks and severe mental illness, respectively), were stricter than current recommendations for treatment of HCV. Indeed, more recent research has identified similar SVR outcomes in a patient population with high rates of mental illness and the presence of continued opioid use (17, 22, 23, 35). Our study utilized a retrospective design. While being treated for HCV was associated with less illicit substance use and higher retention rates in care, a prospective study design would be needed to assess whether there is a causal relationship. Our investigation was also limited to a single site and a homogenous patient demographic. In the site that provided the intervention, eligible participants were offered the opportunity to meet with the HCV NP based on the single provider's capacity. How many people were offered the opportunity and reasons for any decline to participate were not recorded. These data should be recorded in future studies to understand the uptake of integrated treatment and potential barriers to participation. It is possible that individuals who chose to participate in this intervention could be different than those who declined to participate in the intervention. Future research should include baseline comparison of social and medical constructs. There were significant missing data regarding race and ethnicity in the electronic health record, so we do not know the extent to which this sample mirrors general population of people affected by OUD and HCV. There were, however, no differences between groups based on age, gender, or race (among patients for whom racial identity is available). Still, the results should be interpreted with caution, and may not be generalizable to racial and ethnic minority subgroups. Finally, only a few patients were being treated with naltrexone for extended-release injectable, so comparisons between naltrexone and buprenorphine could not be made. Though the study provided a real-world patient experience, a future multi-site study could allow for a more diverse sample of participants, including those being treated with medications for opioid use disorder other than buprenorphine (e.g., methadone, naltrexone) and other preparations of buprenorphine (i.e., injectable).

### Implications

This study of HCV treatment integrated within a commercial outpatient opioid treatment clinic has significant potential implications for the health system and policy. Efforts to achieve an 80% reduction in HCV globally by 2030 (13) will fall short unless screening and treatment of individuals seeking care for OUD is policy focus. Even an incremental increase (10%) in HCV treatment with DAA in areas with typical HCV prevalence (60%), has the potential to make a dramatic impact (20). Our treatment model was carried out by an advanced practice registered nurse with additional training specific to the treatment of HCV, which is currently not an option in every state. Simpler treatment algorithms since the introduction of DAA have made it more feasible for advanced practice registered nurses, physician's assistants, pharmacists, and those outside of specialty care (e.g., primary care) to provide treatment for HCV (36, 37). Similarly, patients face administrative barriers such as lengthy prior authorization procedures (29), sobriety requirements, and limitations on the number of treatment courses covered (37); easing these unnecessary restrictions would increase access to care.

We have reported on a reproducible intervention that lends itself to other outpatient treatment settings. Our analyses have demonstrated the potential positive that integrated HCV treatment may have on patient recovery, including reduction in illicit opioid use, reduced cocaine use, and increased retention in OUD care. Based on these data, HCV care is being expanded to 72 additional [*Treatment Program*] sites across 10 states within our national network of outpatient addiction clinics, and we encourage other treatment clinics to consider offering integrated HCV and MOUD treatments as well.

## Conclusion

Treating and curing HCV in patients seeking care for OUD is vital to achieving elimination of HCV. These data suggest that scaling up integration of HCV treatment in a commercial outpatient OUD treatment clinic is feasible, effective and may improve recovery outcomes.

## Data availability statement

The datasets presented in this article are not readily available because data for this study cannot be shared as it has been obtained from an outpatient OUD treatment organization that has restricted any further sharing of the data. Additional information on use of the data should be directed to LC, lchiodo@addictionref.org. Requests to access the datasets should be directed to lchiodo@addictionref.org.

## Ethics statement

The protocol was reviewed at Northeastern University and deemed not human subjects research.

## Author contributions

PL, SM, and AW conceptualized the study and participated in writing and revision of the manuscript. PL implemented the intervention. LC and JB conducted statistical analysis and interpretation and participated in writing and revision of the manuscript. All authors have reviewed and approved the submitted manuscript.

## Conflict of interest

PL is employed by CleanSlate Outpatient Addiction Medicine and receives speaker's fees from Gilead Sciences, Inc. AW has equity in CleanSlate Outpatient Addiction Medicine. The remaining authors declare that the research was conducted in the absence of any commercial or financial relationships that could be construed as a potential conflict of interest.

## Publisher's note

All claims expressed in this article are solely those of the authors and do not necessarily represent those of their affiliated organizations, or those of the publisher, the editors and the reviewers. Any product that may be evaluated in this article, or claim that may be made by its manufacturer, is not guaranteed or endorsed by the publisher.
